# Routine piloting in systematic reviews—a modified approach?

**DOI:** 10.1186/2046-4053-3-77

**Published:** 2014-07-18

**Authors:** Linda Long

**Affiliations:** 1 Peninsula Technology Assessment Group (PenTAG), University of Exeter Medical School, Veysey Building, Salmon Pool Lane, Exeter, Devon, England EX2 4SG, UK

**Keywords:** Systematic review, Methodology, Piloting, Validity, Efficiency, Data extraction, Pilot systematic review

## Abstract

**Background:**

A continuous growth in the publication of research papers means that there is an expanding volume of data available to the systematic reviewer. Sometimes, researchers can become overwhelmed by the sheer volume of data being processed, leading to inefficient data extraction. This paper seeks to address this problem by proposing a modification to the current systematic review methodology.

**Proposed method:**

This paper details the routine piloting of a systematic review all the way through to evidence-synthesis stage using data from a sample of included papers.

**Results and discussion:**

The result of piloting a sample of papers through to evidence-synthesis stage is to produce a ‘mini systematic review’. Insights from such a pilot review may be used to modify the criteria in the data extraction form. It is proposed that this approach will ensure that in the full review the most useful and relevant information is extracted from all the papers in one phase without needing to re-visit the individual papers at a later stage.

**Conclusions:**

Routine piloting in systematic reviews has been developed in response to advances in information technology and the subsequent increase in rapid access to clinical papers and data. It is proposed that the routine piloting of large systematic reviews will enable themes and meaning in the data to become apparent early in the review process. This, in turn, will facilitate the efficient extraction of data from all the papers in the full review. It is proposed that this approach will result in increased validity of the review, with potential benefits for increasing efficiency.

## Background

The aim of research synthesis, or systematic reviewing, is to arrive at a more comprehensive and trustworthy picture of the topic being studied than is possible from individual pieces of research [1]. Systematic reviewing is a relatively young and rapidly developing field of study, with current best practice defined by guidelines such as those from the Centre for Reviews and Dissemination in the University of York and the Cochrane Handbook [2,3]. However, people embarking on systematic reviews still have many challenges to overcome, particularly relating to resource constraints. Reviews are major outputs of research and require time and resources, and tensions often arise when conducting a methodologically rigorous systematic review with a tightly defined timeline or budget [4].

A continuous growth in the publication of research papers, together with advances in the ability to identify published reports of controlled trials and information about on-going and unpublished studies in registers, means that there is a constantly expanding volume of research papers available [5-7]. Exhaustive searching can reveal high numbers of potentially relevant papers, and each paper must be processed and, if eligible, data extracted and assessed. Researchers might need to extract large volumes of data from the original articles during the data extraction stage. Sometimes, researchers can become overwhelmed by the sheer volume of data being processed. Often, it is not until the final synthesis stage (when data are fully tabulated or examined as a forest plot) that the patterns and relationships across the extracted data emerge, and the implications of the findings from each study, and from the review as a whole, become fully understood. At this late stage, researchers might realise that they have not extracted all the information they need in order to fully answer their research question and will need to repeat earlier steps. On the other hand, they may realise that the data they have spent considerable time extracting is not in fact required. Either way, time (and money) may be lost by inefficient extraction of the relevant data.

This paper seeks to address this problem by proposing a modification to the current systematic review methodology, to take account of the rising volumes of clinical trial data available now and increasingly in the future.

### Main text

At present, it is standard practice to pilot various stages in the systematic review process. Piloting of search strategies (‘scoping searches’), piloting of inclusion/exclusion criteria (within the screening stage) and piloting of data extraction tables or quality assessment checklists are all regularly employed by systematic reviewers [3]. In order to maximise efficiency and minimise error when conducting large systematic reviews, this paper proposes extending such piloting to include all stages of the review process. In essence, this paper proposes conducting a ‘mini systematic review’, all the way through to evidence synthesis, on a sample of included studies. Insights from such a synthesis may be used to inform and refine data extraction in the full systematic review. It is proposed that such a routine modification of the systematic review process will result in an overall decrease in error and time to completion of the full review, even when time for piloting is taken into consideration. Another benefit for undertaking piloting across the entire review process may be that unforeseen problems with a review may be highlighted early on in the review process. This could then lead to a timely re-visiting and modification of the scope, the question or the ambition of the review, and may even support well-informed decisions to stop working on some reviews.

This paper proposes to routinely pilot the systematic review process all the way through to evidence synthesis using data from a sample of included papers, but stopping short of the interpretation of the data to prepare implications for future practice and research. Extracted data from the sample papers will be processed up to the synthesis stage before embarking on the full systematic review. Insights from the pilot synthesis stage may be used to modify criteria in the data extraction form, ensuring that only information needed to answer the review question is extracted from all the included studies. Such a modification will ensure that the most useful and relevant information is extracted from all the papers in a single phase without needing to re-visit the individual papers at a later stage. The proposed modification to the standard methodology hence ensures that resources are more effectively managed, in order to help ensure that the review question is answered within time and budget.

### Proposed method

Traditionally, a detailed review protocol which clearly states the question to be addressed, the subgroups of interest and the methods and criteria to be employed for identifying and selecting relevant studies and extracting and analysing information, is prepared in advance [3,8]. This is important to avoid bias being introduced by decisions that are influenced by the accumulating data. Piloting of data extraction tables and quality appraisal forms is also routinely used to ensure that the information to be extracted is both standardised and relevant. It is common practice to take a small sample (e.g. 10) of included papers and use these to pilot data extraction and quality appraisal. This paper proposes an extension to current systematic review methodology, in which a full ‘trial run’ (or pilot) of the review would be done on such a sample of papers, resulting in a ‘mini systematic review’.

It is proposed that for a review with 20 or more included studies, the method would require a purposive sample of up to 10 papers. These 10 papers would be selected from the pool of included trials by the review authors and would be specifically chosen to cover a wide range of parameters for the review, such as outcome measure scales and/or time points, found in the literature of interest. By taking this sample of papers through each stage of the review process, we can estimate, at an early stage, the potential of the extracted data to answer the review question. Data extraction forms may then be refined and amended following the sample scoping exercise, ensuring efficient data extraction of all the studies to be included in the review.

**Figure 1:**
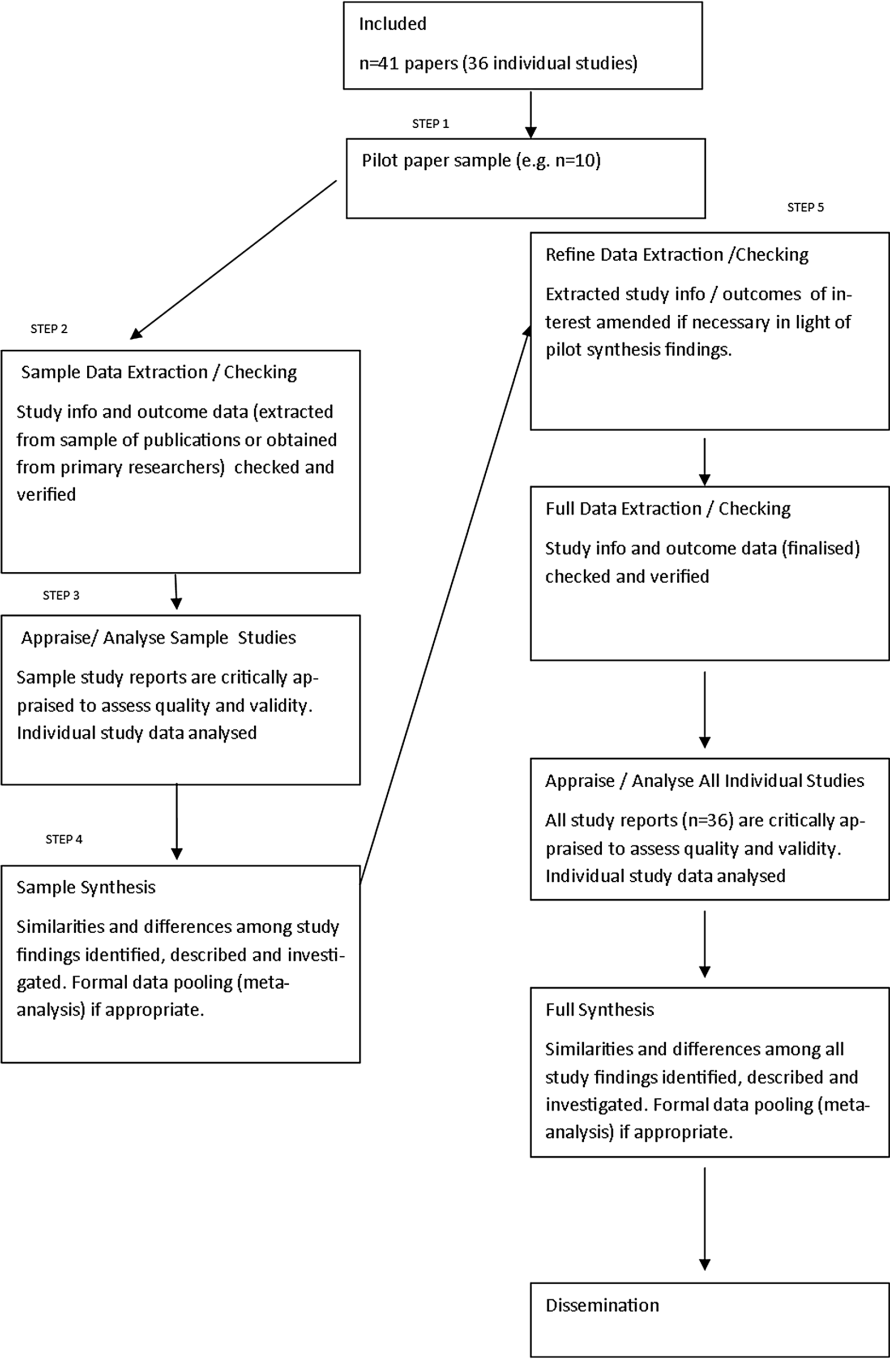
Theoretical process model for the pilot method.

Specifically, it is proposed that current guidelines in conducting systematic reviews are followed up to the stage where papers have been assessed for inclusion in the review [2,3]. The pilot method is then employed, and a purposive sample (reporting a wide range of outcome measure scales and time points) of included papers is chosen from the total number of included studies to pilot up to the synthesis stage (step 1). Data from the sample papers are extracted (step 2) and critically appraised for quality and validity (step 3), and a sample synthesis is then performed (step 4). Results from this pilot synthesis are then used to inform modification of data extraction forms in the light of these preliminary synthesis findings (step 5), to ensure efficient and meaningful extraction of data from all included papers. By documenting the stages clearly in a study flow diagram (see Figure 1), the proposed methodological amendment is systematic, explicit and reproducible, thus minimising data dredging (where a mass of trial data is tested *post hoc* in order to find significant results and associations) and reducing bias. Strengths and weaknesses of such an approach are given in Table 1, and a theoretical case study of how the piloting process may be usefully employed is given below:

**Table 1:** Strengths and weaknesses of piloting systematic reviews prior to full review

**Advantages**	**Weaknesses**
Efficient and potentially time-saving when processing large numbers of studies	Not necessary for reviews with small numbers of included studies.
Greater flexibility for optimally efficient data extraction	Need to have access to most of the potentially eligible studies in order to draw the purposive sample. If the gathering of studies for a review is taking place over a long time period, e.g. months, it might not be possible to identify the sample to start with.
Can be used in large umbrella overviews (a ‘review of systematic reviews’)	Not necessary for overviews with small numbers of included systematic reviews.

#### Theoretical case study

A systematic review of adults with type 2 diabetes exploring the association between biomedical outcomes (e.g. HbA1c, BMI/weight and blood pressure) and quality of life (including low mood and depression) following a diabetic drug intervention. Extraction of quantitative data would be performed using a structured data extraction form to include key study details, patient characteristics, diabetes-related factors, intervention, setting and outcome measures. It may emerge through the piloting process that in addition to baseline diagnosis of depression, a patient's history of depression is important in predicting changes in blood glucose levels (as measured by HbA1c), and so, the data extraction form would need to be modified to extract history of depression data from all the review papers. It may also emerge during the piloting process that some studies record single follow-up points for biomedical outcome measures, while others have multiple follow-up time points. After consideration of the pilot synthesis stage, the data extraction form could be modified to ensure that only the most clinically relevant time points required to answer the review question are extracted in the full review.

## Discussion

This paper describes a new process for routine piloting within large systematic reviews. It is proposed that the method offers a timely evolution of established systematic review methodology. It has been developed in response to advances in information technology and the subsequent increase in rapid access to clinical papers and data. The need to efficiently process increasing volumes of clinical trial data is set to continue into the future, and it is proposed that the routine piloting of large systematic reviews will enable patterns and relationships in the data to become apparent early in the review process. This, in turn, will facilitate the efficient extraction of data from all the papers in the full review. It is proposed that this approach will result in increased external validity of the review, with potential benefits for increasing efficiency, particularly for systematic review teams who have methodological expertise, but no clinical experience of the health intervention under review. Systematic reviewers involved in health technology assessment (HTA) work, where researchers regularly review different clinical areas, may particularly benefit from this approach. In addition, the method may be beneficial if undertaking a systematic review where consideration of generalizability of randomised trials is of particular interest [9]. This may be particularly important where the review teams have concerns about the potential lack of generalizability of included trials. In such reviews, efficient extraction of data on the intervention process and context may benefit most from a rapid assessment early in the review process, in order for a comprehensive and meaningful analysis to be undertaken. Cochrane systematic review teams tend to have strong expertise in the clinical area they are reviewing and are hence more able to define *a priori* the outcomes and scales of most interest to both the patient and the clinician. It is hence likely that there may not be such a need to use the routine piloting method to increase review validity. However, the possibility remains that Cochrane teams engaged in a large-scale review or ‘review of reviews’ may benefit from the method in order to increase efficiency of data extraction. Evaluation of the method in different contexts is hence needed.

Routine piloting of systematic reviews is one response to the need for increased efficiency in an age of increasing access to clinical trial data. Other approaches to increase efficiency involve semantic technologies to automate part of the review process and include text mining (The Evidence for Policy and Practice Information and Co-ordinating Centre (EPPI-Centre)) [10], Systematic Review Data Repositories [11,12] and automation of the production of (Cochrane) protocols [13,14]. More recently, the complete automation of systematic reviews has been proposed [4,13,15]. While offering tremendous potential benefits for increased efficiency, there are some concerns relating to interpretation of language and extrapolation of meaning in fully automated reviews [16,17]. Interpreting data is a crucial aspect in a review, and critics of automation in systematic reviews state that human judgement and interpretation are irreplaceable in order to derive meaning from the evidence synthesised and draw clinically relevant conclusions, especially from pooled data [16]. In addition, human involvement is necessary in order to contact authors to assess if a study meets the inclusion criteria of a review and for the evaluation of risk of bias when reporting of original studies is not complete or precise [16-18]. Others point out that given the often inconsistent and incomplete descriptions of public health interventions, interpretation plays a crucial role when re-describing, classifying or quantifying health interventions, and this might best be done by humans [19,20].

## Conclusions

Routine full piloting of systematic reviews may offer a timely addition to the systematic reviewer's methodology toolkit, whereby human engagement in the review process ensures that meaningful interpretation of results and hence validity (clinical relevance) of the review are maximised, while efficiency is potentially increased. In addition to potentially maximising review validity and efficiency, the method might be usefully employed in emerging areas of systematic review methodology, e.g. ‘reviews of reviews’ and generalizability of randomised controlled trials, where clear and detailed methodological guidance is yet to be fully established.

It is hoped that this paper will serve as stimulation for further discourse on the subject of maximising validity and efficiency in systematic reviews, given the increasing volume of research papers available both now and in the future. Details of approaches developed by other research teams to address this issue, or evaluation of the above routine piloting method, would be most welcome.

## Abbreviations

HTA: Health technology assessment; EPPI-Centre: The Evidence for Policy and Practice Information and Co-ordinating Centre.

## Competing interests

The author declares that she has no competing interests.

## Authors’ information

Dr. L Long is a Research Fellow at the University of Exeter Medical School where she currently works reviewing clinical evidence for health technology assessments, particularly to support the work of NICE.
